# Aripiprazole Facilitates Extinction of Conditioned Fear in Adolescent Rats

**DOI:** 10.3389/fnbeh.2017.00076

**Published:** 2017-05-09

**Authors:** Despina E. Ganella, Liubov Lee-Kardashyan, Sophia J. Luikinga, Danny L. D. Nguyen, Heather B. Madsen, Isabel C. Zbukvic, Russell Coulthard, Andrew J. Lawrence, Jee Hyun Kim

**Affiliations:** ^1^Behavioral Neuroscience Division, Florey Institute of Neuroscience and Mental HealthParkville, VIC, Australia; ^2^Florey Department of Neuroscience and Mental Health, University of MelbourneParkville, VIC, Australia

**Keywords:** adolescence, dopamine, extinction, fear, aripiprazole, prefrontal cortex

## Abstract

Anxiety disorders are the most common type of mental disorder during adolescence, which is at least partly due to the resistance to extinction exhibited at this age. The dopaminergic system is known to be dysregulated during adolescence; therefore, we aimed to facilitate extinction in adolescent rats using the dopamine receptor 2 partial agonist aripiprazole (Abilify™), and examine the behavioral and neural outcomes. Adolescent rats were conditioned to fear a tone. The next day, rats received extinction 30 min after a systemic injection of either 5 mg/kg aripiprazole or vehicle, and then were tested the following day. For the immunohistochemistry experiment, naïve and “no extinction” conditions were added and rats were perfused either on the extinction day or test day. To assess the activation of neurons receiving dopaminergic input, c-Fos, and dopamine- and cAMP-regulated neuronal phosphoprotein (DARPP-32) labeled neurons were quantified in the amygdala and the medial prefrontal cortex (mPFC). Systemic treatment with aripiprazole at the time of extinction significantly reduced freezing at test the next day. This effect was not observed in rats that were fear conditioned but did not receive any extinction. Aripiprazole's facilitation of extinction was accompanied by increased activation of neurons in the mPFC. Taken together, aripiprazole represents a novel pharmacological adjunct to exposure therapy worthy of further examination. The effect of aripiprazole is related to enhanced activation of mPFC neurons receiving dopaminergic innervation.

## Introduction

Adolescence has been repeatedly identified as a vulnerable period for the prevalence of anxiety disorders (Merikangas et al., 2010b; Polanczyk et al., 2015), with adolescent onset of anxiety disorders being a strong predictor for anxiety later in life (Roza et al., 2003). We have previously demonstrated that adolescent rats are impaired in extinction of conditioned fear compared to preadolescent and adult rats (Kim et al., 2011), a finding that has since been replicated in humans (Pattwell et al., 2012). Extinction refers to how fear to a stimulus can be reduced by repeated presentations of that stimulus without any adverse consequences. It is the process that underlies exposure-based therapies integral to cognitive-behavioral therapy. Thus, our rodent studies directly model how adolescents are more resistant to treatments of this nature and are more likely to relapse compared to other ages (Southam-Gerow et al., 2001; Kim and Ganella, 2015).

Our present aim is to investigate a drug that may facilitate extinction in adolescent rats in order to discover an effective pharmacological adjunct to behavioral therapy. While behavioral therapies are the most effective way to treat anxiety disorders, less than one in five adolescents have received them for their anxiety (Merikangas et al., 2011). In fact, anxiety disorders show the biggest gap between prevalence and treatment rates out of all types of youth mental disorders (Merikangas et al., 2010a). This was identified in part due to financial costs and accessibility of behavioral therapy, which is often more expensive and time consuming compared to medication (Merikangas et al., 2010a, 2011). We propose that an effective pharmacological adjunct that facilitates exposure therapy would significantly reduce the amount of treatment necessary, cost, and chronic use of medication, and increase the accessibility of services for more anxious adolescents.

The dopaminergic system plays a key role in extinction processes, and therefore offers a potential target to enhance learning and memory that occur during exposure therapy (Pezze and Feldon, 2004; Peters et al., 2009; Abraham et al., 2014). Importantly, there is a well-established imbalance between dopamine receptor 1 vs. 2 (D1R vs. D2R) signaling during adolescence, which leads to inefficiency in prefrontal cortical processing that is required for cue inhibition (Andersen et al., 2000; Kim et al., 2017). Specifically, there is a relative dominance of D1R compared to D2R activity in the medial prefrontal cortex (mPFC) during adolescence compared to adulthood (Tarazi et al., 1998; Seamans and Yang, 2004). As such, we chose to enhance D2R signaling during extinction using aripiprazole, which is predominantly a D2R partial agonist. Indeed, we have recently shown that extinction of a cue previously associated with cocaine is significantly facilitated by a pre-extinction systemic injection of aripiprazole in adolescent rats (Zbukvic et al., 2016). Interestingly, the pharmacological profile of aripiprazole is not limited to activity at dopamine receptors, it is also a partial agonist at the serotonin 5HT_1A_ receptor, and partial antagonist at 5HT_2A_ (Jordan et al., 2002; DeLeon et al., 2004). Considering that a recent study demonstrated that 5HT_1A_ receptor agonism may facilitate fear extinction via release of dopamine into cortical regions (Saito et al., 2013), testing aripiprazole's potential effects on fear extinction appear warranted.

Aripiprazole is a Food and Drug Administration (FDA) approved medication used widely in teenagers for neuropsychiatric conditions, such as schizophrenia (Burris et al., 2002; Jordan et al., 2002; Davies et al., 2004; DeLeon et al., 2004). It has good tolerability and a low side effect profile making it an ideal candidate for use as an adjunct to treatment of anxiety disorders (DeLeon et al., 2004). Therefore, we examined aripiprazole's potential to facilitate fear extinction in adolescent rats, and assessed associated activation of the dopamine innervated neurons in the mPFC and the amygdala by measuring the immediate early gene c-Fos (Dragunow and Faull, 1989) and dopamine- and cAMP regulated neuronal phosphoprotein (DARPP-32; Hemmings et al., 1984; Gould and Manji, 2005).

## Materials and methods

### Subjects

Male Sprague-Dawley rats were used for the present study (bred in-house). Rats were weaned at postnatal day (P) 21, and were housed with littermates in groups of 6 in individually ventilated cages under a 12/12 h cycle (lights on: 07:00) with food and water available *ad libitum*. All rats were P34 (±1) at the start of experimentation and handled for 3 days prior. All animals were treated in accordance with the guidelines for animal use set out in the Australian code of practice for the care and use of animals for scientific purposes (8th edition, 2013), and all procedures were approved by the Animal Care and Ethics Committee of the Florey Institute of Neuroscience and Mental Health, Melbourne, Australia.

### Apparatus

Behavioral experiments were conducted using *Contextual Near Infra-red Fear Conditioning System* and *Video Freeze* system (Med Associates, VT, USA). The dimensions of the chambers were as described previously (Ganella et al., 2016), with the grid floor composed of 19 × 4.8 mm stainless steel rods that delivered scrambled electric shocks as needed. The chambers were located in two individual rooms to provide two different contexts (Context A and Context B) for conditioning and extinction, as described previously (Ganella et al., 2016). Briefly, Context A had houselights on, round stickers on the back wall with wood chip bedding beneath the grid floor, and cleaned with soap containing a mild eucalyptus odor. Context B had curved walls, and a tray of paper towel placed beneath the grid floor, and cleaned with 80% v/v ethanol.

### Drug injections

Aripiprazole (Alliaance Biotech, India) was suspended in a solution of 5% v/v Tween-80 (Sigma-Aldrich Co., MO, USA) in saline at a concentration of 5 mg/ml. Vehicle was 5% v/v Tween-80 in saline. All rats were injected subcutaneously at a volume of 1 ml/kg. Our dose of 5 mg/kg was chosen based on previous studies examining systemic injections of aripiprazole (Feltenstein et al., 2007; Zbukvic et al., 2016).

### Procedures

*Conditioning*. Rats were placed in the novel experimental chambers without receiving prior habituation, and after a 2 min baseline period, the conditioned stimulus (CS, tone; 5000 Hz, 80 dB) was presented for 10 s, which co-terminated with the 1 s unconditioned stimulus (US, foot-shock; 0.6 mA). Rats received 3 CS-US pairings. The inter-trial interval (ITI) ranged between 85 and 135 s, with a mean of 110 s.

*Extinction*. The next day, rats were injected with aripiprazole or vehicle. Thirty minutes following an injection, the rat was placed in a different context to fear conditioning. After a 2 min baseline period the 10 s CS was presented alone 30 times (10 s ITI). Rats that received “no extinction” (Experiment 2) were placed in the chamber for the identical period of time as extinguished rats without any CSs. In Experiment 2, half of the rats were perfused 90 min following extinction while the remaining rats were tested the next day. *Test*. The day after extinction, rats were tested back in the extinction context (ABB design), with baseline freezing recorded in the first minute, followed by a 2 min presentation of the CS. The remaining rats in Experiment 2 were perfused 90 min following test.

### Perfusions

Rats were terminally anesthetized with sodium pentobarbital (100 mg/kg, i.p.), then transcardially perfused with 50 ml 0.1 M phosphate buffered saline (PBS) followed by ~250 ml 4% paraformaldehyde (PFA) in 0.1 M PBS. The perfused brains were post-fixed for 1 h in PFA, washed in PBS for 1 h, and then left overnight in a 20% sucrose PBS solution. Brains were then snap frozen using liquid nitrogen and stored at −20°C until sectioned.

### Tissue processing and immunohistochemistry

Coronal slices of the mPFC and amygdala were cut in a 1 in 4 series at a thickness of 40 μm and stored in cryoprotectant at −20°C until immunohistochemistry. Sections were washed 3 × 10 min in PBS, then incubated for 30 min in a blocking solution (10% normal donkey serum (NDS) and 0.5% Triton X-100 in PBS). Brain tissue was then incubated overnight at 21°C with primary antibodies: rabbit anti-DARPP-32 at 1:1000 (AB10518, Merck Millipore, Massachusetts, USA) and goat anti-c-Fos at 1:500 (sc-52-G, Santa Cruz Biotechnology, Texas, USA) in PBS with 1% NDS and 0.5% Triton X-100. Sections were then washed in PBS, blocked (60 min), and then incubated for 2 h in the dark at 21°C in secondary antibodies: donkey anti-rabbit IgG at 1:200 (Alexa Fluor 488, Life Technologies, CA, USA) and donkey anti-goat IgG at 1:200 (Alexa Fluor 594, Life Technologies, CA, USA) in PBS with 1% NDS and 0.5% Triton X-100. Sections were then washed in PBS prior to mounting onto gelatin coated slides, and coverslipped with fluorescent mounting medium (Dako North America Inc., CA, USA).

### Microscopy and quantification of immunohistochemistry

We imaged and counted 3 rostrocaudal sections per region in the right hemisphere (+3.2 to +2.88 mm for the mPFC and −2.7 to −3.02 mm for the amygdala; DM LB2 microscope, Leica Microsystems, North Ryde, Australia) as described previously (Kim et al., 2012). Because DARPP-32 levels did not vary across any groups, DARPP-32/c-Fos double labeling was standardized as a percentage of total DARPP-32 immunostaining (%DARPP-DBL). Images from the mPFC were cropped as individual prelimbic (e.g., PrL) or IL regions, and amygdala images were cropped into subregions, central amygdala (e.g., CeA), basal amygdala (BA) and lateral amygdala (LA) for manual counting, which were delineated into standardized area size according to the rat brain atlas (Paxinos and Watson, 1998). Observers unaware of experimental groups counted using ImageJ (NIH, MD, USA).

### Data analyses

In all behavioral sessions, freezing was measured as the fear conditioned response (CR) to the CS, which was calculated via automated near infra-red video tracking equipment and computer software (Video Freeze, Med Associates, VT, USA) with a motion threshold 50, as described previously (Handford et al., 2014). For all experiments, conditioning and extinction data were analyzed using repeated-measures (RM) analysis of variance (ANOVA), with *p* ≤ 0.05. There were no significant differences in baseline freezing for any experiments on conditioning, extinction or test days (*p*s > 0.05), see Table 1. For analyses of immunohistochemical data we included the counts per rostrocaudal section as a within-subjects factor, and have reported the rostrocaudal effects only for those that were significantly affected by experimental manipulation (i.e., Extinction Condition and/or Drug) in the present results. Two brains from post-test perfusions were severely damaged during freezing and we were unable to count c-Fos and DARPP immunostaining (No Extinction—Vehicle *n* = 1; Extinction—Vehicle *n* = 1).

**Table 1:** **Mean levels of percent baseline freezing at extinction and test (mean ± standard error of the mean)**.

**Experiment**	**Group**	***n***	**Extinction**	**Test**
1	Vehicle	8	5.5 (± 2.3)	31.4 (± 16.7)
	Aripiprazole	9	14.9 (± 11.2)	21.1 (± 11.2)
2	No Extinction—Vehicle	12	4.7 (± 6.1)	2.7 (± 1.8)
	No Extinction—Aripiprazole	13	6.0 (± 2.4)	1.3 (± 1.2)
	Extinction—Vehicle	12	33.2 (± 10.8)	18 (± 10.8)
	Extinction—Aripiprazole	12	4.2 (± 8.6)	2.3 (± 2.0)

*There were no significant group differences in either experiment (ps > 0.05)*.

## Results

### Experiment 1—pre-extinction systemic injection of aripiprazole facilitates long-term extinction in adolescent rats

**Figure 1:**
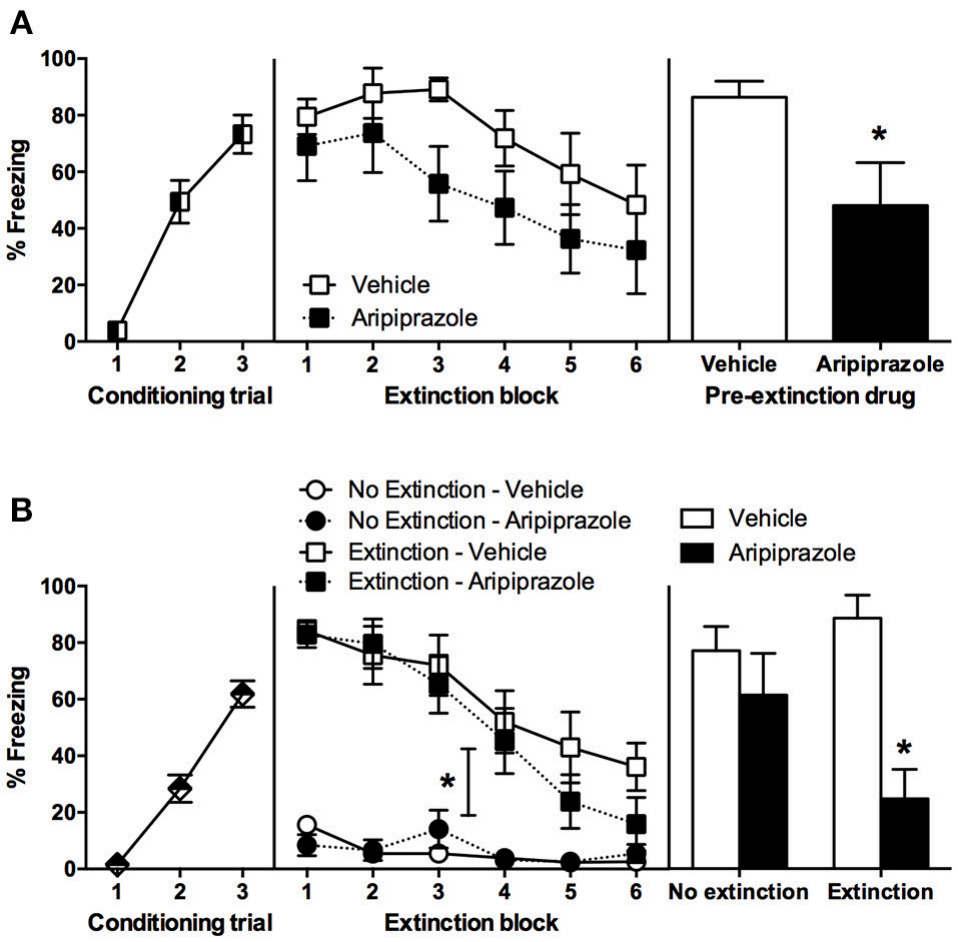
**Mean (± standard error of the mean) levels of conditioned stimulus (CS)-elicited freezing. (A)** Experiment 1, rats were conditioned on day 1, received extinction 30 min after rats received an injection of either aripiprazole (5 mg/kg) or vehicle on day 2, and then tested for CS-elicited freezing on day 3. Aripiprazole *n* = 9; Vehicle *n* = 8. “^*^” Indicates a significant effect of drug (*p* < 0.05). **(B)** Experiment 2, rats were conditioned on day 1, there were no differences between groups so all data was pooled. On day 2, rats receiving aripiprazole (5 mg/kg) or vehicle underwent CS-extinction or were exposed to the chamber with no CS presentations, “no extinction” 30 min after injection (Extinction–Aripiprazole *n* = 12; Extinction–Vehicle *n* = 12; No Extinction–Aripiprazole *n* = 13; No Extinction–Vehicle *n* = 12). “^*^” Indicates a significant interaction between Extinction Condition and Extinction Block (*p* < 0.05). Some rats were perfused 90 min after extinction (Extinction–Aripiprazole *n* = 7; Extinction–Vehicle *n* = 6; No Extinction–Aripiprazole *n* = 7; No Extinction–Vehicle *n* = 6); the remaining rats were tested for CS-elicited freezing the next day and then perfused 90 min after test (Extinction–Aripiprazole *n* = 5; Extinction–Vehicle *n* = 6; No Extinction–Aripiprazole *n* = 6; No Extinction–Vehicle *n* = 6). “^*^” Indicates a significant *post-hoc* effect of drug (*p* < 0.05) following a significant drug × extinction interaction.

Rats fear conditioned comparably, with a significant effect of CS-US Trial [*F*_(2, 30)_ = 44.2, *p* < 0.0001] but no other effects (*p*s > 0.05), therefore the conditioning data are pooled across pre-extinction drug condition in Figure 1A. On extinction day there was a significant effect of extinction Block [*F*_(5, 75)_ = 4.5, *p* < 0.01], but no main effect of drug [*F*_(1, 15)_ = 3.9, *p* = 0.07] and no other effects (*p*s > 0.05). In order to test whether long-term extinction was effective in adolescent rats, we conducted RM ANOVA comparing the last block of extinction and test. This yielded a significant effect of day [*F*_(1, 15)_ = 5.5, *p* < 0.05], which indicates that adolescent rats failed to show long-term extinction in this experiment. At test there was a significant effect of Drug [*t*_(15)_ = 2.3 *p* < 0.05], indicating that pre-extinction injection of aripiprazole significantly facilitated long-term extinction in adolescent rats.

### Experiment 2—aripiprazole effects are specific following cue extinction, and lead to activation of the prelimbic cortex neurons at test

There were no significant differences between groups in conditioning; therefore conditioning data from all rats were pooled (Figure 1B). During extinction, there was a significant effect of Extinction Block [*F*_(5, 225)_ = 37.8, *p* < 0.0001], Extinction Condition [*F*_(1, 45)_ = 77.4, *p* < 0.0001], and an interaction between the two [*F*_(5, 225)_ = 19.5, *p* < 0.0001], but no other effects (*ps* > 0.05). These results indicate that only the rats in the extinction condition froze and then extinguished to CS, while the no extinction rats that were merely exposed to the chamber did not freeze. In order to test whether long-term extinction was effective, we conducted RM ANOVA comparing the last block of extinction and test in rats that received extinction. This yielded a significant effect of day [*F*_(1, 9)_ = 10.7, *p* < 0.05], which indicates that adolescent rats failed to show long-term extinction. At test there was a significant effect of Drug [*F*_(1, 19)_ = 13.3, *p* < 0.01], and an interaction between Extinction Condition × Drug [*F*_(1, 19)_ = 4.9, *p* < 0.05], but no effect of Extinction Condition (*p* > 0.05). *Post-hoc t*-tests revealed that in extinction groups there was a significant effect of Drug [*t*_(9)_ = 4.9, *p* < 0.001], with no such effect in the no extinction groups (*t* < 1). Taken together, extinction in adolescent rats did not reduce freezing levels at test compared to no extinction controls. Systemic injection of aripiprazole prior to extinction alleviated this extinction deficit and significantly reduced freezing at test.

**Figure 2:**
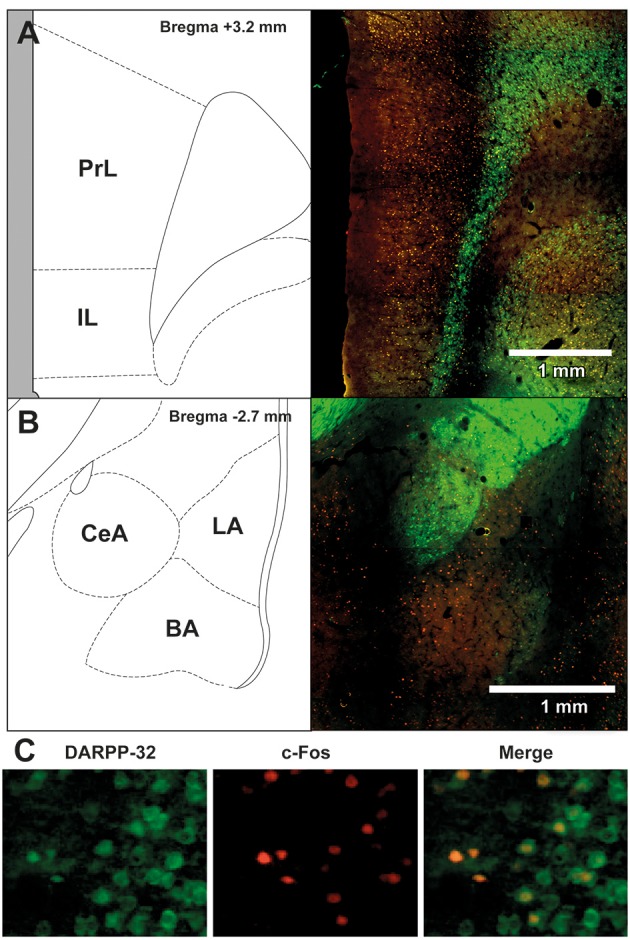
**Representative images of the immunohistochemical staining for DARPP-32 positive (green) and c-Fos positive cells (red) in (A)** medial prefrontal cortex (mPFC), including the prelimbic (PrL) and infralimbic (IL) regions, and **(B)** amygdala, including the central amygdala (CeA), basal amygdala (BA) and lateral amygdala (LA) subregions. The left panels show the anatomical schemactic taken from the rat atlas (Paxinos and Watson, 1998) that was used to define boundaries of individual subregions within the mPFC and amygdala. **(C)** Representative image taken from a rat in the extinction—aripiprazole group, of DARPP-32 positive cells (left panel, green), c-Fos positive cells (middle panel, red) and merge (right panel) showing cells that are co-labeled with c-Fos and DARPP-32.

**Figure 3:**
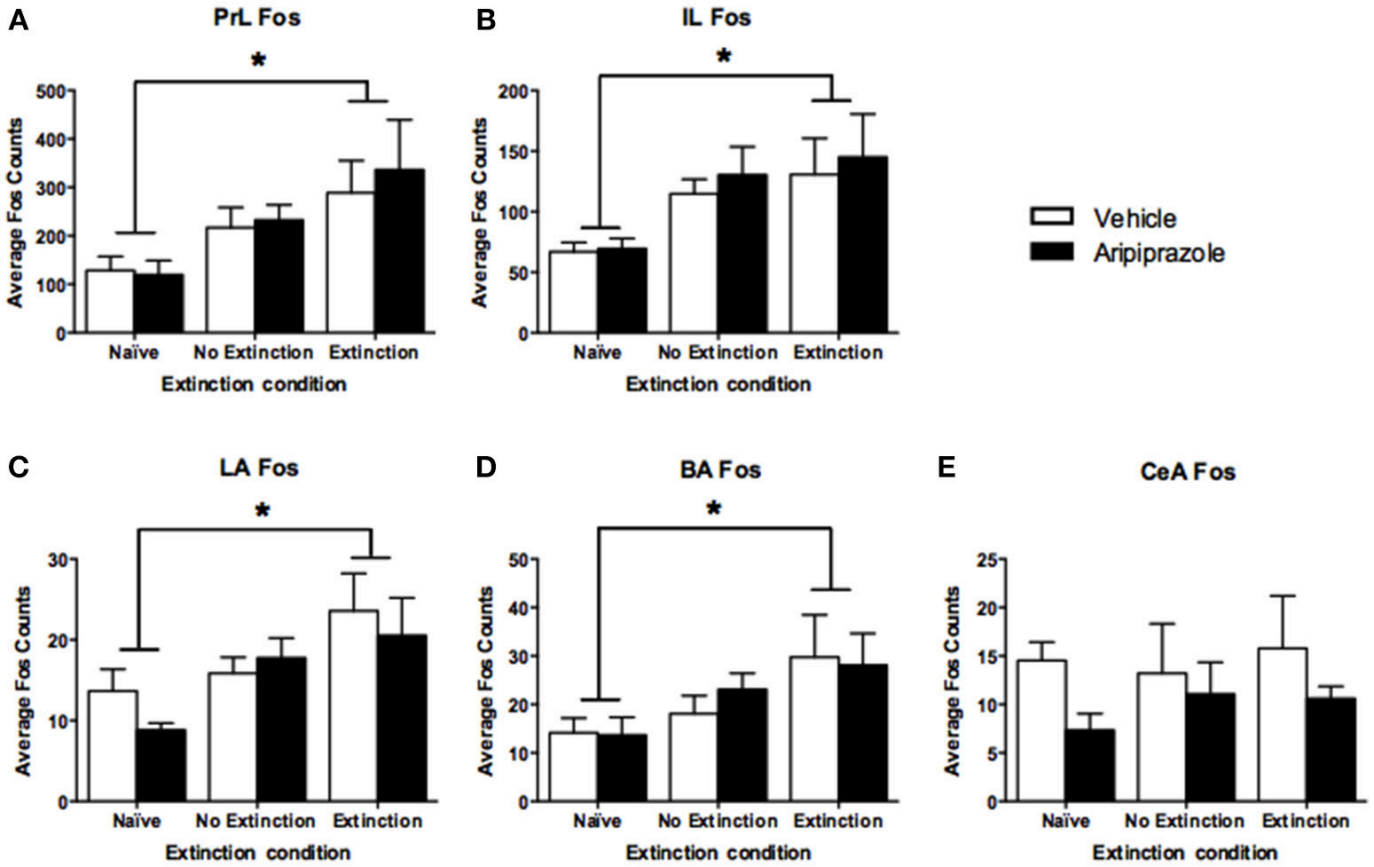
**Mean (± standard error of the mean) c-Fos counts across three rostrocaudal sections of the medial prefrontal cortex and amygdala of rats perfused post-extinction. (A)** prelimbic region (PrL), **(B)** infralimbic region (IL), **(C)** lateral amygdala (LA), **(D)** basal amygdala (BA), and **(E)** central amygdala (CeA). Extinction–Aripiprazole *n* = 7; Extinction–Vehicle *n* = 6; No Extinction–Aripiprazole *n* = 7; No Extinction–Vehicle *n* = 6; Naïve–Aripiprazole *n* = 4; Naïve–Vehicle *n* = 4. “^*^” Indicates a significant effect of extinction condition (naïve vs. extinction) following *post-hoc* tests with Tukey multiple comparisons (*p* < 0.05).

To assess the neural effects of pre-extinction aripiprazole following extinction or test, an additional group of “naïve” rats were included for immunohistochemistry. Naïve rats were handled alongside the rats that underwent behavior, and were injected with either aripiprazole or vehicle 30 mins before being handled, then perfused 90 min post-handling. We examined c-Fos as a marker for neuronal activation (Dragunow and Faull, 1989), and DARPP-32 as a marker for dopaminergic input (Hemmings et al., 1984; Gould and Manji, 2005). See Figure 2 for representations of brain regions and staining. In the brains perfused post-extinction, there were significant effects of Extinction Condition of PrL-Fos [*F*_(2, 28)_ = 3.6, *p* < 0.05], IL-Fos [*F*_(2, 28)_ = 3.2, *p* < 0.05], LA-Fos [*F*_(2, 28)_ = 4.1, *p* < 0.05], and a trend for BA-Fos [*F*_(2, 28)_ = 3.1, *p* = 0.059], but no other main effects or interactions in DARPP-32 counts or double-labeling (Table 2). *Post-hoc* tests with Tukey multiple comparisons revealed a significant difference between rats in the naïve vs. extinction condition for PrL-Fos, IL-Fos, BA-Fos, and LA-Fos (*ps* < 0.05; Figure 3).

**Table 2:** **DARPP immunolabeled cells and DARPP/Fos double labeled cells as a percentage of total DARPP staining (%DARPP-DBL) in the prefrontal cortex and amygdala of rats perfused post-extinction**.

**Experimental condition**	**Brain region**	**DARPP (**± **SEM)**	**% DARPP-DBL (**± **SEM)**
Naïve—vehicle	PrL	339.0 ± 41.7	5.0 ± 3.3
	IL	125.2 ± 14.9	8.4 ± 4.2
	CeA	67.4 ± 19.9	7.2 ± 3.9
	BA	29.3 ± 16.9	16.0 ± 11.3
	LA	11.6 ± 3.3	30.1 ± 10.1
Naïve—aripiprazole	PrL	403.9 ± 66.3	3.5 ± 1.5
	IL	112.9 ± 24.7	10.1 ± 5.8
	CeA	93.4 ± 17.4	3.7 ± 0.9
	BA	48.0 ± 13.7	12.3 ± 5.8
	LA	12.2 ± 3.1	10.9 ± 7.6
No Extinction—vehicle	PrL	379.7 ± 37.0	5.9 ± 1.5
	IL	137.3 ± 18.0	9.2 ± 2.0
	CeA	83.5 ± 12.4	5.3 ± 2.3
	BA	79.2 ± 14.6	8.4 ± 2.6
	LA	21.3 ± 4.1	30.1 ± 9.2
No extinction—aripiprazole	PrL	419.0 ± 41.8	9.8 ± 3.1
	IL	140.3 ± 15.0	15.3 ± 5.1
	CeA	84.4 ± 15.0	3.5 ± 1.2
	BA	70.6 ± 14.3	13.4 ± 11.1
	LA	24.2 ± 3.9	20.0 ± 9.7
Extinction—vehicle	PrL	408.1 ± 44.9	9.3 ± 2.5
	IL	160.9 ± 24.5	10.4 ± 2.9
	CeA	71.9 ± 5.4	8.6 ± 1.8
	BA	84.1 ± 12.4	9.3 ± 5.6
	LA	17.7 ± 1.9	28.5 ± 13.1
Extinction—aripiprazole	PrL	438.0 ± 34.4	11.0 ± 2.5
	IL	148.4 ± 12.0	12.9 ± 2.4
	CeA	75.6 ± 11.1	8.7 ± 3.2
	BA	85.0 ± 20.8	10.6 ± 3.3
	LA	19.8 ± 5.0	25.2 ± 7.8

*Values are represented as a group mean of the average counts across three rostrocaudal sections imaged ± SEM. PrL, prelimbic cortex; IL, infralimbic cortex; CeA, central amygdala; BA, basal amygdala; LA, lateral amygdala; DARPP, dopamine- and cAMP- regulated phosphoprotein; SEM, standard error of the mean*.

**Figure 4:**
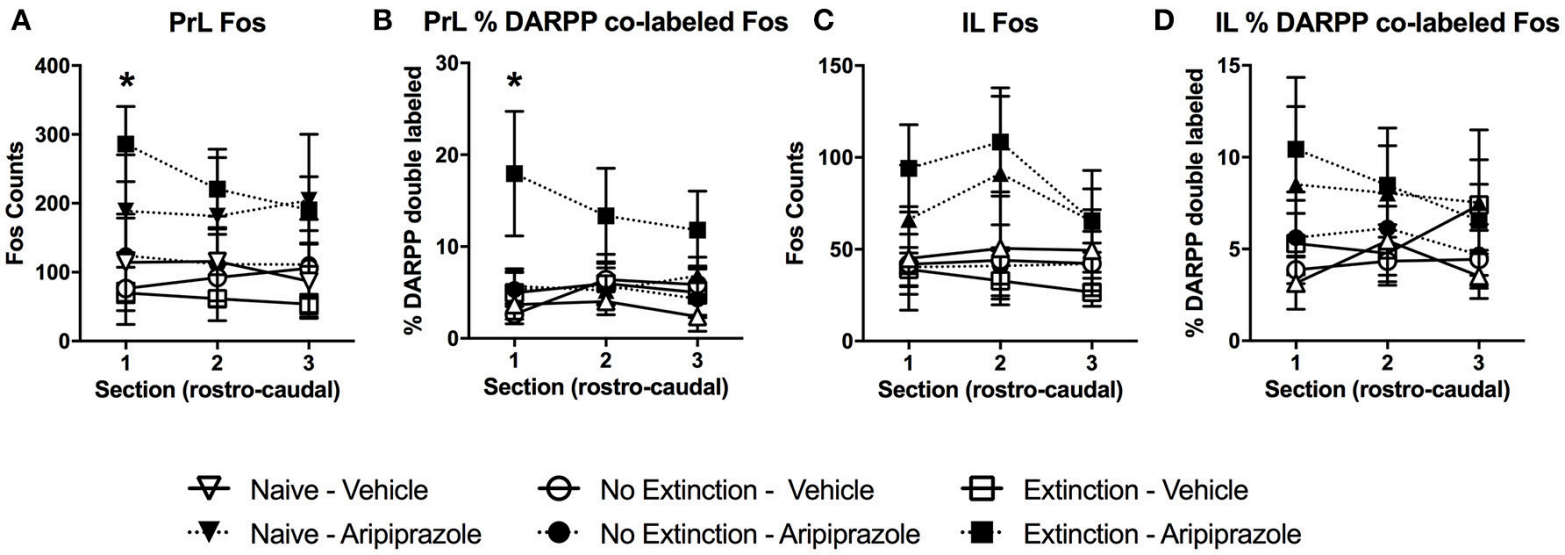
**Mean (± standard error of the mean) of c-Fos and DARPP/Fos double labeled cells as a percentage of total DARPP staining (%DARPP-DBL) across three individual rostrocaudal sections of the medial prefrontal cortex of rats perfused post-test. (A)** Prelimbic region (PrL) c-Fos staining, **(B)** PrL %DARPP co-labeled with Fos, **(C)** infralimbic region (IL) c-Fos staining, and **(D)** IL %DARPP co-labeled with Fos. Extinction–Aripiprazole *n* = 5; Extinction–Vehicle *n* = 5; No Extinction–Aripiprazole *n* = 6; No Extinction–Vehicle *n* = 5; Naïve–Aripiprazole *n* = 4; Naïve–Vehicle *n* = 4. “^*^” Indicates a significant effect of Drug for that section with *post-hoc* analysis (*p* < 0.05).

In the brains perfused post-test, there were no effects in DARPP-32 counts (Tables 3 and 4 for average DARPP-32 and Fos counts). There was an Extinction Condition × Section interaction identified for PrL-Fos [*F*_(4, 46)_ = 7.1, *p* < 0.001], PrL-%DARPP-DBL [*F*_(4, 46)_ = 3.1, *p* < 0.05] and IL-Fos [*F*_(4, 46)_ = 3.0, *p* < 0.05]. There was a Drug × Section interaction for PrL-Fos [*F*_(2, 46)_ = 4.9, *p* < 0.05], PrL-%DARPP-DBL [*F*_(2, 46)_ = 4.5, *p* < 0.05], IL-Fos [*F*_(2, 46)_ = 3.6, *p* < 0.05] and IL-%DARPP-DBL [*F*_(2, 46)_ = 4.1, *p* < 0.05]. *Post-hoc* analysis for each section revealed that there was a significant effect of Drug for PrL1-Fos, [*F*_(1, 23)_ = 6.00, *p* < 0.05] and PrL1-%DARPP-DBL, [*F*_(1, 23)_ = 4.6, *p* < 0.05], but no effects in IL-Fos or IL-%DARPP-DBL (*p*s > 0.05; Figure 4).

**Table 3:** **DARPP immunolabeled cells and DARPP/Fos double labeled cells as a percentage of total DARPP staining (%DARPP-DBL) in the prefrontal cortex and amygdala of rats perfused post-test**.

**Experimental condition**	**Brain region**	**DARPP (± SEM)**	**%DARPP-DBL (± SEM)**
Naïve–vehicle	PrL	527.4 ± 104.1	3.4 ± 1.6
	IL	146.0 ± 32.6	4.0 ± 1.6
	CeA	130.1 ± 23.4	1.5 ± 0.5
	BA	39.5 ± 15.8	6.0 ± 3.7
	LA	26.2 ± 11.7	2.0 ± 2.3
Naïve–aripiprazole	PrL	600.6 ± 86.1	5.9 ± 2.0
	IL	157.8 ± 34.4	6.7 ± 2.1
	CeA	155.8 ± 23.6	2.1 ± 0.9
	BA	119.3 ± 27.8	2.4 ± 1.0
	LA	27.3 ± 5.9	1.8 ± 1.4
No Extinction–vehicle	PrL	529.6 ± 62.4	5.0 ± 2.1
	IL	222.3 ± 17.9	4.2 ± 1.2
	CeA	119.2 ± 17.7	3.0 ± 1.5
	BA	92.5 ± 9.8	5.3 ± 2.4
	LA	29.9 ± 4.2	7.8 ± 3.5
No extinction–aripiprazole	PrL	492.9 ± 56.1	5.1 ± 1.9
	IL	150.2 ± 14.4	5.5 ± 2.0
	CeA	341.0 ± 53.6	1.7 ± 0.9
	BA	90.0 ± 29.2	3.0 ± 1.1
	LA	19.8 ± 5.4	6.6 ± 1.9
Extinction–vehicle	PrL	455.9 ± 51.6	5.3 ± 2.7
	IL	176.9 ± 32.2	5.8 ± 1.9
	CeA	142.2 ± 27.3	1.5 ± 0.5
	BA	89.6 ± 12.9	10.0 ± 4.6
	LA	20.9 ± 1.4	13.5 ± 10.9
Extinction–aripiprazole	PrL	442.4 ± 56.3	14.4 ± 6.0
	IL	160.3 ± 18.5	8.5 ± 2.3
	CeA	96.1 ± 19.8	10.1 ± 7.0
	BA	57.1 ± 28.1	4.5 ± 2.9
	LA	14.7 ± 3.5	5.3 ± 2.9

*Values are represented as a group mean of the average counts across three rostrocaudal sections imaged ± SEM. PrL, prelimbic cortex; IL, infralimbic cortex; CeA, central amygdala; BA, basal amygdala; LA, lateral amygdala; DARPP, dopamine- and cAMP- regulated phosphoprotein; SEM, standard error of the mean*.

**Table 4:** **Average Fos counts in the prefrontal cortex and amygdala of rats perfused post-test**.

**Experimental condition**	**Brain region**	**Fos (± SEM)**
Naïve–vehicle	PrL	105.7 ± 73.0
	IL	48.3 ± 30.5
	CeA	9.8 ± 3.6
	BA	14.3 ± 7.6
	LA	11.8 ± 6.0
Naïve–aripiprazole	PrL	191.6 ± 100.2
	IL	74.3 ± 37.8
	CeA	22.8 ± 60.3
	BA	60.3 ± 36.0
	LA	34.8 ± 23.3
No Extinction–vehicle	PrL	91.5 ± 69.3
	IL	42.7 ± 19.9
	CeA	12.9 ± 4.9
	BA	46.0 ± 23.0
	LA	30.7 ± 16.7
No extinction–aripiprazole	PrL	115.7 ± 57.3
	IL	41.2 ± 11.3
	CeA	10.9 ± 4.6
	BA	27.1 ± 5.3
	LA	13.7 ± 3.1
Extinction–vehicle	PrL	61.7 ± 13.0
	IL	32.8 ± 8.9
	CeA	5.8 ± 1.6
	BA	18.8 ± 3.4
	LA	12.5 ± 3.2
Extinction–aripiprazole	PrL	232.4 ± 59.5
	IL	89.3 ± 25.5
	CeA	17.9 ± 10.7
	BA	49.2 ± 31.9
	LA	32.5 ± 22.6

*Values are represented as a group mean of the average counts across three rostrocaudal sections imaged ± SEM. PrL, prelimbic cortex; IL, infralimbic cortex; CeA, central amygdala; BA, basal amygdala; LA, lateral amygdala; SEM, standard error of the mean*.

There was also a 3-way interaction between Section × Drug × Extinction Condition for PrL-Fos [*F*_(4, 46)_ = 5.4, *p* < 0.05]. *Post-hoc* analysis for each rostrocaudal section indicated that aripiprazole significantly increased PrL-Fos in the extinction groups [*t*_(8)_ = 9.8 *p* < 0.05], but not in other groups. No effects were found in amygdala regions post-test (*ps* > 0.05).

## Discussion

Our results demonstrate that adolescent rats show impaired long-term extinction that can be ameliorated with a single systemic injection of aripiprazole prior to extinction. As shown in our previous studies, an extinction session involving 30 CS-alone presentations in adolescent rats did not lead to a long-term reduction in fear when tested the next day. When extinction was combined with aripiprazole, however, adolescent rats showed a significant decrease in CS-elicited fear at test. Aripiprazole reduced freezing at test only when given in conjunction with extinction, with no extinction rats maintaining high CS-elicited freezing levels at test. This is important because it shows that aripiprazole does not merely cause non-specific reductions in freezing at test. Aripiprazole also did not significantly affect the rate of within-session extinction or overall levels of freezing during the extinction session, suggesting that it may strengthen the CS-no shock “safety” memory of extinction once acquired. Regardless of the drug received, post-extinction perfused brains showed that extinction induced significant c-Fos labeling in the PrL, IL, LA, and the BA compared to naïve rats, although the no extinction group were not significantly different to either conditions. Because extinction and no extinction groups were placed in a novel chamber compared to the naïve rats that were merely handled, this result suggest that an exposure to a novel chamber may contribute to increases in c-Fos labeling observed following extinction, while novelty alone does not induce significant changes in c-Fos labeling compared to being handled. Interestingly, pre-extinction aripiprazole did not significantly affect the number of c-Fos labeled neurons following extinction in the amygdala or the mPFC, whereas it significantly increased c-Fos labeled neurons in the PrL following test.

### Enhancing extinction in adolescence

Our finding that adolescent rats show ineffective long-term extinction is consistent with the growing literature on rodent findings modeling adolescent vulnerability to relapse in anxiety disorders (McCallum et al., 2010; Kim et al., 2011; Pattwell et al., 2012, 2016; Baker and Richardson, 2015; Zbukvic et al., 2017). Using this rodent model, various manipulations that can alleviate such adolescent impairment in extinction have since been discovered. The first effective behavioral manipulation discovered was doubling the amount of extinction (McCallum et al., 2010; Kim et al., 2011). However, doubling the amount of extinction is impractical and costly in the clinical setting, because exposure therapy can require months to years to complete (Foa and McLean, 2016). The second effective behavioral manipulation discovered was administering extinction in the fear conditioning context (Pattwell et al., 2016). Again, this approach is difficult to translate, as it would be impractical or impossible to conduct exposure therapy where the fear memory was initially formed in anxious adolescents. Consequently, we contend that the best approach that is readily translatable to the clinic would be to facilitate extinction using acute pharmacological adjuncts at the time of extinction to reduce the amount of exposure therapy needed. Indeed, it has been observed that systemic injection of the NMDA receptor partial agonist D-cycloserine (DCS) following extinction can significantly reduce the relapse of extinguished fear observed in adolescent rats (McCallum et al., 2010). DCS is one of the most widely used medications worldwide for the treatment of tuberculosis, and while it has been shown to facilitate extinction robustly in rodents, large-scale clinical trials in humans have reported small to no effects (for review see Otto et al., 2015). In fact, a 2015 Cochrane review found no evidence of DCS efficacy to facilitate exposure therapy (Ori et al., 2015). Additionally, we have recently demonstrated that systemic injection of DCS following fear conditioning can significantly augment fear conditioned responding (Handford et al., 2014), which suggests that administration of DCS too close to a traumatic event may exacerbate the fear. Therefore, the present finding that aripiprazole facilitates extinction in adolescent rats offers a new pharmacological adjunct for further investigation to enhance exposure therapy, at least in adolescents. Interestingly, we have noted previously that while aripiprazole alone failed to promote abstinence in cocaine users, it was effective in cocaine users that also received behavioral therapy in conjunction with aripiprazole (Kim and Lawrence, 2014). Taken together, we believe aripiprazole is a promising candidate for future extinction studies attempting to discover effective pharmacological adjuncts to exposure therapy.

### Neural correlates of extinction in adolescence and effects of aripiprazole

In the present study, reduced freezing at test due to pre-extinction injection of aripiprazole was accompanied by molecular changes specific to the mPFC that were influenced by both drug and extinction condition in brains examined post-test. Our data showed that in the most rostral part of PrL, aripiprazole caused increased activation of neurons, as well as an increase in the proportion of activated neurons receiving dopaminergic input. This is consistent with anterograde tracer studies in adult rats which have shown that injections into rostral PrL lead to dense labeling of neurons in the BLA, suggesting that connections from this part of the PrL are important in mediating the expression of extinguished fear responses (Sesack et al., 1989).

**Figure 5:**
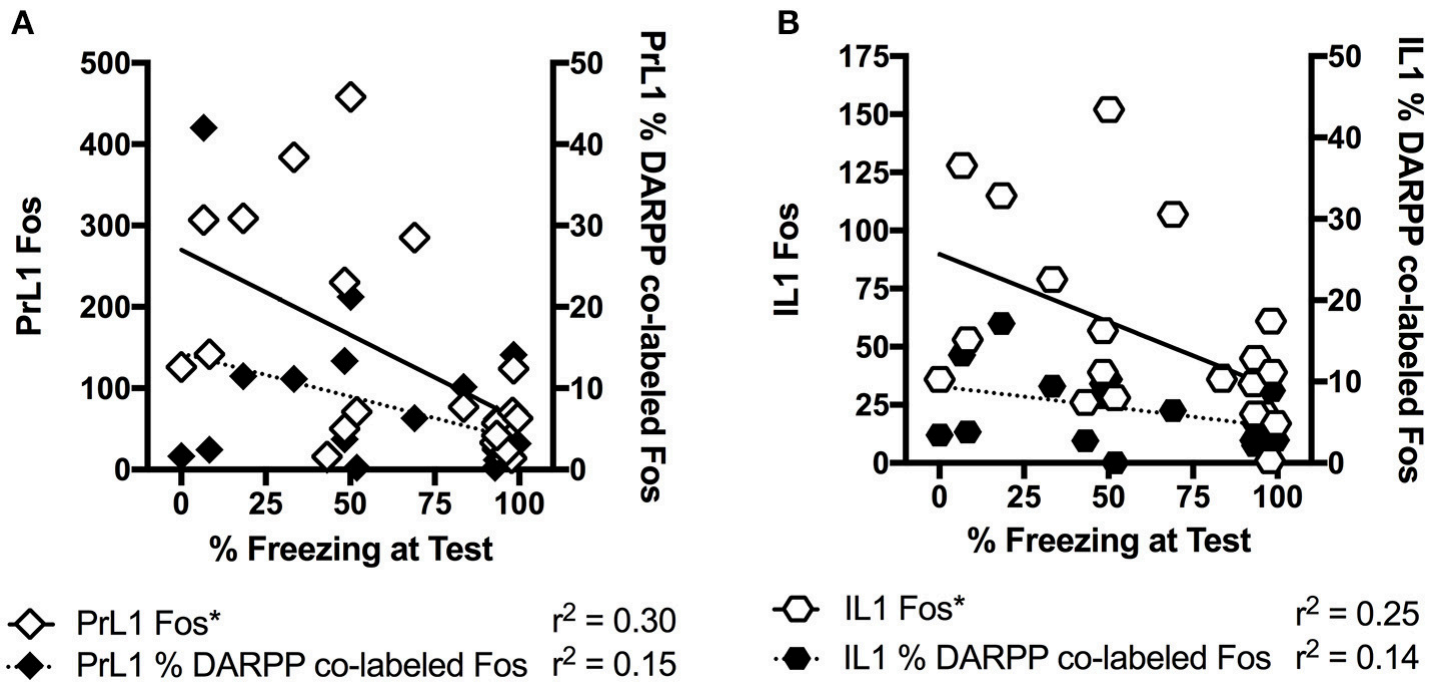
**Correlation analyses for percentage freezing at test with c-Fos staining and percentage of DARPP co-labeled with Fos staining in the rostral section of prelimbic (PrL) cortex (A)** and infralimbic (IL) cortex **(B)**. “^*^” Indicates a significant negative correlation between freezing and Fos expression. All rats that underwent testing were included, *n* = 21.

When we further explored the present findings, freezing levels at test significantly correlated with Fos levels in the rostral PrL (*r* = −0.54, *p* < 0.05), but not with % DARPP-DBL levels (*r* = −0.38, *p* = 0.09) in the rostral PrL (Figure 5). The significant negative correlation indicates that lower freezing levels at test is associated with higher Fos expression in the PrL, which is contrary to the dominant model of extinction expression (Peters et al., 2009). Specifically, studies in adult rodents have demonstrated that the function of PrL and IL are dissociated (Sierra-Mercado et al., 2011), with the IL critical for reducing fear expression, and the PrL critical for fear expression (Milad and Quirk, 2002; Burgos-Robles et al., 2009; Sotres-Bayon et al., 2012; Pattwell et al., 2016). However, the role of IL and PrL in conditioned fear expression is still poorly understood in adolescent rodents. Given that the prefrontal circuit is undergoing rapid structural changes during this developmental stage (Cunningham et al., 2002; Schubert et al., 2015), the roles of IL and PrL in fear expression might not be as dissociated during adolescence compared to adulthood. For example, when we explored correlations between freezing levels at test and the rostral IL from the present study, the results were similar to PrL (c-Fos: *r* = −0.50, *p* < 0.05; %DARPP-DBL: *r* = −0.38, *p* = 0.09; Figure 5). Figure 3 also shows that the pattern of extinction-dependent Fos expression in PrL and IL are similar. Further, in our original findings examining adolescent fear extinction, PrL and IL showed similar patterns of phosphorylated mitogen-activated protein kinase (pMAPK) that were related to amounts of extinction received in adolescent rats (Kim et al., 2011). Indeed, Giustino and Maren recently reviewed the role of the mPFC in the extinction of fear and highlight evidence to challenge the existing models of PrL and IL dissociation (see Giustino and Maren, 2015 for review). They suggest that there is some functional overlap between the two regions, which allows one structure to compensate for the other structure under certain conditions, and adolescence may be one such condition.

Notably, Pattwell and colleagues recently investigated the prefrontal circuitry associated with persistent fear memories during adolescence. They identified selective and dynamic reorganization of synaptic spine circuitry with elevated synaptic spine production within the PrL, but not in the IL, during a developmental period which coincides with resistance to cue extinction learning in adolescent mice (Pattwell et al., 2016). They also observed a selective surge in BLA-PrL but not BLA-IL connectivity during adolescence that correlates with resistance to cued fear extinction (Pattwell et al., 2016). While it is currently unknown which underlying molecular mechanisms drive a selective enhancement in connectivity between the PrL and BLA, it may involve the dopaminergic system and modulation of this system may affect expression of fear during adolescence.

Communication between the amygdala and prefrontal cortex relies on the fine balance between excitatory and inhibitory dopaminergic transmission (Jackson et al., 2001; Floresco and Maric, 2007). During adolescence, there are dramatic changes in dopamine receptor expression in the mPFC, with a transient relative dominance of D1R against D2R activity (Andersen et al., 2000; Kim et al., 2017). This is particularly relevant for the emergence of anxiety disorders; computational models predict PFC D1R signaling to be activated by CS conditioning whereas PFC D2R signaling to be activated by CS extinction (Seamans and Yang, 2004). Consequently, the imbalance of D1R/D2R signaling during adolescence likely contributes to the development of strong fear-associated cues that are inhibition-resistant due to impaired top down control from prefrontal regions to limbic regions. This may contribute to adolescents being more resistant to treatments such as cue-exposure therapy. Interestingly, IL infusion of both D1R and D2R antagonists before extinction have been shown to impair long-term extinction retention in adult rats (Hikind and Maroun, 2008; Mueller et al., 2010). It appears that more studies are necessary to delineate the role of D1R and D2R in fear extinction, especially considering that D1R and D2R signaling has opposite modulation of the intracellular pathways (Seamans and Yang, 2004).

Aripiprazole is thought to stabilize the dopaminergic system by acting on both postsynaptic D2R and presynaptic autoreceptors (DeLeon et al., 2004). In the present study, administration of aripiprazole may have promoted activation of a prefrontal pathway involving D2R that leads to enhanced extinction memory consolidation and/or recall the next day via a mechanism involving activation of prefrontal neurons (Hemmings et al., 1984; Gould and Manji, 2005). Consistent with this idea, aripiprazole has been shown to increase the dopamine pool in the rat PFC (Ratajczak et al., 2016). Also, in healthy adult humans oral administration of aripiprazole led to enhanced dorsolateral PFC (DLPFC) activation associated with a trend for improved discriminability and reaction times compared with placebo (Murphy et al., 2016). Those researchers concluded that aripiprazole has unique DLPFC actions attributed to its prefrontal D2R agonist action. Aripiprazole may be working via a similar mechanism in our study, whereby D2R agonism in neurons of the mPFC, particularly the PrL, may enhance the strength of the extinction memory in adolescent rodents. The changes in prefrontal c-Fos expression observed at test suggest that aripiprazole may affect extinction memory consolidation leading to a better retrieval of extinction memory. Santini and colleagues have shown that the mPFC is a critical site of successful extinction memory consolidation and storage and this is accompanied by increased c-Fos expression in the mPFC as a result of extinction training (Santini et al., 2004).

It is important to note that the mechanism underlying the effects of aripiprazole may not be limited to D2R and may also involve the serotonergic system. While aripiprazole displays robust preferential binding to D2R in both rats (Natesan et al., 2006) and humans (Mamo et al., 2007), it also exhibits partial agonist activity at the serotonin receptor 5HT_1A_ and partial antagonism at 5HT_2A_ (Jordan et al., 2002; DeLeon et al., 2004). In fact the partial antagonism by aripiprazole of the 5HT_2A_ receptor is thought to minimize excessive dopaminergic blockade by increasing dopamine release (Millan, 2003; DeLeon et al., 2004). Both 5HT_1A_ and 5HT_2A_ receptors have been implicated in anxiety related behaviors (Stahl, 2000; Lanzenberger et al., 2007; Fisher et al., 2009; Zhang et al., 2013). For example, Saito et al. showed that the 5HT_1*A*_ receptor agonist, tandospirone, facilitated extinction retrieval, which was accompanied by changes in synaptic function and increased cortical dopamine levels (Saito et al., 2013). The serotonin system has also been shown to strongly mediate prefrontal dopamine signaling and fiber infiltration into the mPFC (Benes et al., 2000) and serotonergic fibers interact with both dopamine afferents and gamma-aminobutyric acidergic interneurons in the mPFC (Taylor and Benes, 1996; Taylor et al., 1998). This is particularly relevant during adolescence as dopaminergic and serotonergic inputs to the PFC increase to peak levels above those seen later in life (Kalsbeek et al., 1988).

### Conclusions

There is currently a major “treatment gap” for adolescents suffering from anxiety disorders. A recent study by Merikangas et al. identified that half of adolescents with severely impairing mental disorders have never received mental health treatment for their symptoms (Merikangas et al., 2011). Barriers to treatment include cost, shortages of mental health specialists for youth and repeated access to services necessary for successful treatments (Merikangas et al., 2011). It has been established that cognitive behavioral therapy/exposure based therapies are effective in helping adolescents overcome anxiety, however this requires multiple therapy sessions which may take months or years (Cartwright-Hatton et al., 2004). Reducing the number of therapy sessions necessary with adjunctive pharmacotherapy such as aripiprazole addresses these barriers to treatment, and we encourage future studies to further examine the potential for aripiprazole to promote extinction at various stages of life.

## Author contributions

DG, AL, and JK conceptualized and designed the study. DG, LL, SL, DN, HM, IZ, RC, and JK acquired the data. DG and JK analyzed the data. DG, AL, and JK interpreted the data. DG and JK wrote the manuscript. All authors were involved in critically revising the work for important intellectual content and in final approval of the version to be published. All authors agree to be accountable for all aspects of the work in ensuring that questions related to the accuracy or integrity of any part of the work are appropriately investigated and resolved.

## Funding

This research was supported by a project grant (APP1063140) from the National Health and Medical Research Council (NHMRC) of Australia awarded to JK and AL, a Women In Science Fellowship from the Baker Foundation awarded to DG, Australian Postgraduate Award awarded to IZ, NHMRC Principal Research Fellowship (1020737) awarded to AL, Australian Research Council Discovery Grant (DP150102496) and NHMRC Career Development Fellowship (APP1083309) awarded to JK.

### Conflict of interest statement

The authors declare that the research was conducted in the absence of any commercial or financial relationships that could be construed as a potential conflict of interest.

## References

[B1] Abraham A. D. Neve K. A. Lattal K. M. (2014). Dopamine and extinction: a convergence of theory with fear and reward circuitry. Neurobiol. Learn. Mem. 108, 65–77. 10.1016/j.nlm.2013.11.007 24269353 PMC3927738

[B2] Andersen S. L. Thompson A. T. Rutstein M. Hostetter J. C. Teicher M. H. (2000). Dopamine receptor pruning in prefrontal cortex during the periadolescent period in rats. Synapse 37, 167–169. 10.1002/1098-2396(200008)37:2<167::AID-SYN11>3.0.CO;2-B 10881038

[B3] Baker K. D. Richardson R. (2015). Forming competing fear learning and extinction memories in adolescence makes fear difficult to inhibit. Learn. Mem. 22, 537–543. 10.1101/lm.039487.114 26472643 PMC4749725

[B4] Benes F. M. Taylor J. B. Cunningham M. C. (2000). Convergence and plasticity of monoaminergic systems in the medial prefrontal cortex during the postnatal period: implications for the development of psychopathology. Cereb. Cortex 10, 1014–1027. 10.1093/cercor/10.10.1014 11007552

[B5] Burgos-Robles A. Vidal-Gonzalez I. Quirk G. J. (2009). Sustained conditioned responses in prelimbic prefrontal neurons are correlated with fear expression and extinction failure. J. Neurosci. 29, 8474–8482. 10.1523/JNEUROSCI.0378-09.2009 19571138 PMC2733220

[B6] Burris K. D. Molski T. F. Xu C. Ryan E. Tottori K. Kikuchi T.. (2002). Aripiprazole, a novel antipsychotic, is a high-affinity partial agonist at human dopamine D2 receptors. J. Pharmacol. Exp. Ther. 302, 381–389. 10.1124/jpet.102.033175 12065741

[B7] Cartwright-Hatton S. Roberts C. Chitsabesan P. Fothergill C. Harrington R. (2004). Systematic review of the efficacy of cognitive behaviour therapies for childhood and adolescent anxiety disorders. Br. J. Clin. Psychol. 43, 421–436. 10.1348/0144665042388928 15530212

[B8] Cunningham M. G. Bhattacharyya S. Benes F. M. (2002). Amygdalo-cortical sprouting continues into early adulthood: implications for the development of normal and abnormal function during adolescence. J. Comp. Neurol. 453, 116–130. 10.1002/cne.10376 12373778

[B9] Davies M. A. Sheffler D. J. Roth B. L. (2004). Aripiprazole: a novel atypical antipsychotic drug with a uniquely robust pharmacology. CNS Drug Rev. 10, 317–336. 10.1111/j.1527-3458.2004.tb00030.x 15592581 PMC6741761

[B10] DeLeon A. Patel N. C. Crismon M. L. (2004). Aripiprazole: a comprehensive review of its pharmacology, clinical efficacy, and tolerability. Clin. Ther. 26, 649–666. 10.1016/S0149-2918(04)90066-5 15220010

[B11] Dragunow M. Faull R. (1989). The use of c-fos as a metabolic marker in neuronal pathway tracing. J. Neurosci. Methods 29, 261–265. 10.1016/0165-0270(89)90150-7 2507830

[B12] Feltenstein M. W. Altar C. A. See R. E. (2007). Aripiprazole blocks reinstatement of cocaine seeking in an animal model of relapse. Biol. Psychiatry 61, 582–590. 10.1016/j.biopsych.2006.04.010 16806092

[B13] Fisher P. M. Meltzer C. C. Price J. C. Coleman R. L. Ziolko S. K. Becker C.. (2009). Medial prefrontal cortex 5-HT2A density is correlated with amygdala reactivity, response habituation, and functional coupling. Cereb. Cortex 19, 2499–2507. 10.1093/cercor/bhp022 19321655 PMC2758681

[B14] Floresco S. B. Maric T. T. (2007). Dopaminergic regulation of inhibitory and excitatory transmission in the basolateral amygdala–prefrontal cortical pathway. J. Neurosci. 27, 2045–2057. 10.1523/JNEUROSCI.5474-06.2007 17314300 PMC6673549

[B15] Foa E. B. McLean C. P. (2016). The efficacy of exposure therapy for anxiety-related disorders and its underlying mechanisms: the case of OCD and PTSD. Annu. Rev. Clin. Psychol. 12, 1–28. 10.1146/annurev-clinpsy-021815-093533 26565122

[B16] Ganella D. E. Thangaraju P. Lawrence A. J. Kim J. H. (2016). Fear extinction in 17 day old rats is dependent on metabotropic glutamate receptor 5 signaling. Behav. Brain Res. 298, 32–36. 10.1016/j.bbr.2014.12.010 25497704

[B17] Giustino T. F. Maren S. (2015). The role of the medial prefrontal cortex in the conditioning and extinction of fear. Front. Behav. Neurosci. 9:298. 10.3389/fnbeh.2015.00298 26617500 PMC4637424

[B18] Gould T. D. Manji H. K. (2005). DARPP-32: A molecular switch at the nexus of reward pathway plasticity. Proc. Natl. Acad. Sci. U.S.A. 102, 253–254. 10.1073/pnas.0408700102 15632217 PMC544318

[B19] Handford C. E. Tan S. Lawrence A. J. Kim J. H. (2014). The effect of the mGlu5 negative allosteric modulator MTEP and NMDA receptor partial agonist D-cycloserine on Pavlovian conditioned fear. Int. J. Neuropsychopharmacol. 17, 1521–1532. 10.1017/S1461145714000303 24674862

[B20] Hemmings H. C. Greengard P. Tung H. L. Cohen P. (1984). DARPP-32, a dopamine-regulated neuronal phosphoprotein, is a potent inhibitor of protein phosphatase-1. Nature 310, 503–505. 6087160 10.1038/310503a0

[B21] Hikind N. Maroun M. (2008). Microinfusion of the D1 receptor antagonist, SCH23390 into the IL but not the BLA impairs consolidation of extinction of auditory fear conditioning. Neurobiol. Learn. Mem. 90, 217–222. 10.1016/j.nlm.2008.03.003 18442937

[B22] Jackson M. E. Frost A. S. Moghaddam B. (2001). Stimulation of prefrontal cortex at physiologically relevant frequencies inhibits dopamine release in the nucleus accumbens. J. Neurochem. 78, 920–923. 10.1046/j.1471-4159.2001.00499.x 11520912

[B23] Jordan S. Koprivica V. Chen R. Tottori K. Kikuchi T. Altar C. A. (2002). The antipsychotic aripiprazole is a potent, partial agonist at the human 5-HT 1A receptor. Eur. J. Pharmacol. 441, 137–140. 10.1016/S0014-2999(02)01532-7 12063084

[B24] Kalsbeek A. Voorn P. Buijs R. Pool C. Uylings H. (1988). Development of the dopaminergic innervation in the prefrontal cortex of the rat. J. Comp. Neurol. 269, 58–72. 10.1002/cne.902690105 3361004

[B25] Kim J. H. Ganella D. E. (2015). A review of preclinical studies to understand fear during adolescence. Austr. Psychol. 50, 25–31. 10.1111/ap.12066

[B26] Kim J. H. Lawrence A. J. (2014). Drugs currently in Phase II clinical trials for cocaine addiction. Expert Opin. Investig. Drugs 23, 1105–1122. 10.1517/13543784.2014.915312 24773297

[B27] Kim J. H. Li S. Hamlin A. S. McNally G. P. Richardson R. (2012). Phosphorylation of mitogen-activated protein kinase in the medial prefrontal cortex and the amygdala following memory retrieval or forgetting in developing rats. Neurobiol. Learn. Mem. 97, 59–68. 10.1016/j.nlm.2011.09.005 21963362

[B28] Kim J. H. Li S. Richardson R. (2011). Immunohistochemical analyses of long-term extinction of conditioned fear in adolescent rats. Cereb. Cortex 21, 530–538. 10.1093/cercor/bhq116 20576926

[B29] Kim J. H. Perry C. J. Ganella D. E. Madsen H. B. (2017). Postnatal development of neurotransmitter systems and their relevance in extinction of conditioned fear. Neurobiol. Learn. Mem. 138, 252–270. 10.1016/j.nlm.2016.10.018 27818267

[B30] Lanzenberger R. R. Mitterhauser M. Spindelegger C. Wadsak W. Klein N. Mien L.-K.. (2007). Reduced serotonin-1A receptor binding in social anxiety disorder. Biol. Psychiatry 61, 1081–1089. 10.1016/j.biopsych.2006.05.022 16979141

[B31] Mamo D. Graff A. Mizrahi R. C. M. Shammi M. Romeyer F. Kapur S. (2007). Differential effects of aripiprazole on D 2, 5-HT 2, and 5-HT 1A receptor occupancy in patients with schizophrenia: a triple tracer PET study. 164, 1411–1417. Am. J. Psychiatry 10.1176/appi.ajp.2007.06091479 17728427

[B32] McCallum J. Kim J. H. Richardson R. (2010). Impaired extinction retention in adolescent rats: effects of D-Cycloserine. Neuropsychopharmacology 35, 2134–2142. 10.1038/npp.2010.92 20592716 PMC3055297

[B33] Merikangas K. R. He J.-P. Brody D. Fisher P. W. Bourdon K. Koretz D. S. (2010a). Prevalence and treatment of mental disorders among US children in the 2001–2004 NHANE. Pediatrics 125(1), 75–81. 10.1542/peds.2008-2598 20008426 PMC2938794

[B34] Merikangas K. R. He J.-P. Burstein M. Swendsen J. Avenevoli S. Case B.. (2011). Service utilization for lifetime mental disorders in US adolescents: results of the National Comorbidity Survey–Adolescent Supplement (NCS-A). J. Am. Acad. Child Adolesc. Psychiatry 50, 32–45. 10.1016/j.jaac.2010.10.006 21156268 PMC4408275

[B35] Merikangas K. R. He M. J.-P. Burstein M. Swanson M. S. A. Avenevoli S. Cui M. L.. (2010b). Lifetime prevalence of mental disorders in US adolescents: Results from the National Comorbidity Study-Adolescent Supplement (NCS-A). J. Am. Acad. Child Adolesc. Psychiatry 49:980. 10.1016/j.jaac.2010.05.017 20855043 PMC2946114

[B36] Milad M. R. Quirk G. J. (2002). Neurons in medial prefrontal cortex signal memory for fear extinction. Nature 420, 70–74. 10.1038/nature01138 12422216

[B37] Millan M. J. (2003). The neurobiology and control of anxious states. Prog. Neurobiol. 70, 83–244. 10.1016/S0301-0082(03)00087-X 12927745

[B38] Mueller D. Bravo-Rivera C. Quirk G. J. (2010). Infralimbic D2 receptors are necessary for fear extinction and extinction-related tone responses. Biol. Psychiatry 68, 1055–1060. 10.1016/j.biopsych.2010.08.014 20926066 PMC2981677

[B39] Murphy A. Dursun S. McKie S. Elliott R. Deakin J. F. W. (2016). An investigation into aripiprazole's partial D2 agonist effects within the dorsolateral prefrontal cortex during working memory in healthy volunteers. Psychopharmacology 233, 1415–1426. 10.1007/s00213-016-4234-9 26900078 PMC4819596

[B40] Natesan S. Reckless G. E. Nobrega J. N. Fletcher P. J. Kapur S. (2006). Dissociation between *in vivo* occupancy and functional antagonism of dopamine D2 receptors: comparing aripiprazole to other antipsychotics in animal models. Neuropsychopharmacology 31, 1854–1863. 10.1038/sj.npp.1300983 16319908

[B41] Ori R. Amos T. Bergman H. Soares-Weiser K. Ipser J. C. Stein D. J. (2015). Augmentation of cognitive and behavioural therapies (CBT) with d-cycloserine for anxiety and related disorders. Cochrane Library. 10:CD007803 10.1002/14651858.cd007803.pub2 PMC8939046 25957940

[B42] Otto M. W. Kredlow M. A. Smits J. A. Hofmann S. G. Tolin D. F. de Kleine R. A.. (2015). Enhancement of psychosocial treatment with d-cycloserine: models, moderators, and future directions. Biol. Psychiatry 80, 274-83. 10.1016/j.biopsych.2015.09.007 26520240 PMC4808479

[B43] Pattwell S. S. Duhoux S. Hartley C. A. Johnson D. C. Jing D. Elliott M. D.. (2012). Altered fear learning across development in both mouse and human. Proc. Natl. Acad. Sci. U.S.A. 109, 16318–16323. 10.1073/pnas.1206834109 22988092 PMC3479553

[B44] Pattwell S. S. Liston C. Jing D. Ninan I. Yang R. R. Witztum J.. (2016). Dynamic changes in neural circuitry during adolescence are associated with persistent attenuation of fear memories. Nature communications 7:11475. 10.1038/ncomms11475 27215672 PMC4890178

[B45] Paxinos G. Watson C. (1998). The Rat Brain Atlas in Stereotaxic Coordinates. San Diego, CA: Academic.

[B46] Peters J. Kalivas P. W. Quirk G. J. (2009). Extinction circuits for fear and addiction overlap in prefrontal cortex. Learn. Mem. 16, 279–288. 10.1101/lm.1041309 19380710 PMC4527308

[B47] Pezze M. A. Feldon J. (2004). Mesolimbic dopaminergic pathways in fear conditioning. Prog. Neurobiol. 74, 301–320. 10.1016/j.pneurobio.2004.09.004 15582224

[B48] Polanczyk G. V. Salum G. A. Sugaya L. S. Caye A. Rohde L. A. (2015). Annual research review: a meta-analysis of the worldwide prevalence of mental disorders in children and adolescents. J. Child Psychol. Psychiatry 56, 345–365. 10.1111/jcpp.12381 25649325

[B49] Ratajczak P. Kus K. Gołembiowska K. Noworyta-Sokołowska K. Woźniak A. Zaprutko T.. (2016). The influence of aripiprazole and olanzapine on neurotransmitters level in frontal cortex of prenatally stressed rats. Environ. Toxicol. Pharmacol. 46, 122–130. 10.1016/j.etap.2016.07.007 27458700

[B50] Roza S. J. Hofstra M. B. van der Ende J. Verhulst F. C. (2003). Stable prediction of mood and anxiety disorders based on behavioral and emotional problems in childhood: a 14-year follow-up during childhood, adolescence, and young adulthood. Am. J. Psychiatry 160, 2116–2121. 10.1176/appi.ajp.160.12.2116 14638580

[B51] Saito Y. Matsumoto M. Yanagawa Y. Hiraide S. Inoue S. Kubo Y.. (2013). Facilitation of fear extinction by the 5-HT1A receptor agonist tandospirone: possible involvement of dopaminergic modulation. Synapse 67, 161–170. 10.1002/syn.21621 23152167

[B52] Santini E. Ge H. Ren K. de Ortiz S. P. Quirk G. J. (2004). Consolidation of fear extinction requires protein synthesis in the medial prefrontal cortex. J. Neurosci. 24, 5704–5710. 10.1523/JNEUROSCI.0786-04.2004 15215292 PMC6729226

[B53] Schubert D. Martens G. Kolk S. (2015). Molecular underpinnings of prefrontal cortex development in rodents provide insights into the etiology of neurodevelopmental disorders. Mol. Psychiatry 20, 795–809. 10.1038/mp.2014.147 25450230 PMC4486649

[B54] Seamans J. K. Yang C. R. (2004). The principal features and mechanisms of dopamine modulation in the prefrontal cortex. Prog. Neurobiol. 74, 1–58. 10.1016/j.pneurobio.2004.05.006 15381316

[B55] Sesack S. R. Deutch A. Y. Roth R. H. Bunney B. S. (1989). Topographical organization of the efferent projections of the medial prefrontal cortex in the rat: An anterograde tract-tracing study with Phaseolus vulgaris leucoagglutinin. J. Comp. Neurol. 290, 213–242. 10.1002/cne.902900205 2592611

[B56] Sierra-Mercado D. Padilla-Coreano N. Quirk G. J. (2011). Dissociable roles of prelimbic and infralimbic cortices, ventral hippocampus, and basolateral amygdala in the expression and extinction of conditioned fear. Neuropsychopharmacology 36, 529–538. 10.1038/npp.2010.184 20962768 PMC3005957

[B57] Sotres-Bayon F. Sierra-Mercado D. Pardilla-Delgado E. Quirk G. J. (2012). Gating of fear in prelimbic cortex by hippocampal and amygdala inputs. Neuron 76, 804–812. 10.1016/j.neuron.2012.09.028 23177964 PMC3508462

[B58] Southam-Gerow M. A. Kendall P. C. Weersing V. R. (2001). Examining outcome variability: correlates of treatment response in a child and adolescent anxiety clinic. J. Clin. Child Psychol. 30, 422–436. 10.1207/S15374424JCCP3003_13 11501258

[B59] Stahl S. M. (2000). Essential Psychopharmacology: Neuroscientific Basis and Practical Applications. Cambridge: Cambridge University Press.

[B60] Tarazi F. I. Tomasini E. C. Baldessarini R. J. (1998). Postnatal development of dopamine D 4-like receptors in rat forebrain regions: Comparison with D 2-like receptors. Dev. Brain Res. 110, 227–233. 10.1016/S0165-3806(98)00111-4 9748595

[B61] Taylor J. B. Benes F. M. (1996). Colocalization of glutamate decarboxylase, tyrosine hydroxylase and serotonin immunoreactivity in rat medial prefrontal cortex. Neurosci. Net 1:10001.

[B62] Taylor J. B. Cunningham M. C. Benes F. M. (1998). Neonatal raphe lesions increase dopamine fibers in prefrontal cortex of adult rats. Neuroreport 9, 1811–1815. 10.1097/00001756-199806010-00026 9665606

[B63] Zbukvic I. C. Ganella D. E. Perry C. J. Madsen H. B. Bye C. R. Lawrence A. J.. (2016). Role of Dopamine 2 Receptor in Impaired Drug-Cue Extinction in Adolescent Rats. Cereb. Cortex 26, 2895–2904. 10.1093/cercor/bhw051 26946126 PMC4869820

[B64] Zbukvic I. C. Park J. C. Ganella D. E. Lawrence A. J. Kim J. H. (2017). Prefrontal dopaminergic mechanisms of fear extinction across adolescence. Front. Behav. Neurosci. 11:32. 10.3389/fnbeh.2017.00032 28275342 PMC5319962

[B65] Zhang G. Ásgeirsdóttir H. N. Cohen S. J. Munchow A. H. Barrera M. P. Stackman R. W. (2013). Stimulation of serotonin 2A receptors facilitates consolidation and extinction of fear memory in C57BL/6J mice. Neuropharmacology 64, 403–413. 10.1016/j.neuropharm.2012.06.007 22722027 PMC3477617

